# A spheroid toxicity assay using magnetic 3D bioprinting and real-time mobile device-based imaging

**DOI:** 10.1038/srep13987

**Published:** 2015-09-14

**Authors:** Hubert Tseng, Jacob A. Gage, Tsaiwei Shen, William L. Haisler, Shane K. Neeley, Sue Shiao, Jianbo Chen, Pujan K. Desai, Angela Liao, Chris Hebel, Robert M. Raphael, Jeanne L. Becker, Glauco R. Souza

**Affiliations:** 1 Nano3D Biosciences (n3D), Houston, TX 77030 USA; 2 LC Sciences, Houston, TX 77054 USA; 3 Department of Physics, Rice University, Houston, TX 77005 USA; 4 Department of Bioengineering, Rice University, Houston, TX 77005 USA

## Abstract

An ongoing challenge in biomedical research is the search for simple, yet robust assays using 3D cell cultures for toxicity screening. This study addresses that challenge with a novel spheroid assay, wherein spheroids, formed by magnetic 3D bioprinting, contract immediately as cells rearrange and compact the spheroid in relation to viability and cytoskeletal organization. Thus, spheroid size can be used as a simple metric for toxicity. The goal of this study was to validate spheroid contraction as a cytotoxic endpoint using 3T3 fibroblasts in response to 5 toxic compounds (all-trans retinoic acid, dexamethasone, doxorubicin, 5′-fluorouracil, forskolin), sodium dodecyl sulfate (+control), and penicillin-G (−control). Real-time imaging was performed with a mobile device to increase throughput and efficiency. All compounds but penicillin-G significantly slowed contraction in a dose-dependent manner (Z’ = 0.88). Cells in 3D were more resistant to toxicity than cells in 2D, whose toxicity was measured by the MTT assay. Fluorescent staining and gene expression profiling of spheroids confirmed these findings. The results of this study validate spheroid contraction within this assay as an easy, biologically relevant endpoint for high-throughput compound screening in representative 3D environments.

A major challenge in biomedical research and drug development is predicting *in vivo* responses to drugs *in vitro*. While most *in vitro* assays are cheaper and easier to perform than animal testing, they are often poorly representative of physiological environments^1^,^2^,^3^,^4^,^5^,^6^,^7^. *In vitro* assays are typically performed as two-dimensional (2D) cell monolayers with rigid substrates and unidirectional biochemical concentration gradients that inadequately mimic tissue environments found *in vivo*^3^,^4^,^5^,^6^. Three-dimensional (3D) *in vitro* systems address the shortcomings of 2D systems, offering *in vivo*-like environments and cell-cell and cell-extracellular matrix (ECM) interactions that are crucial to the regulation of cell behavior and function but are difficult to replicate in 2D^8^,^9^. There are many 3D cell culture systems currently available, such as ECM protein gels, hydrogels, and commonly spheroid technologies, like hanging drop cultures, round bottom or micropatterned plates^10^,^11^,^12^,^13^. However, these systsems suffer from various technical challenges that limit their potential. These platforms require long formation times (more than three days to form) and difficult handling that limit high-throughput screening, are difficult to image, particularly with round bottom plates and hanging drop spheroids, and some cell types may not readily generate spheroids^14^,^15^,^16^. Furthermore, technical issues with light penetration and reagent diffusion have made assaying spheroids difficult^17^. These shortcomings in 3D environments necessitate the development of simple, yet robust endpoints for high-throughput toxicity screening.

Towards that end, this study investigated a novel spheroid assay for toxicity screening. The basis of this assay is magnetic 3D bioprinting (M3DB), wherein cells are magnetized with a magnetic nanoparticle assembly consisting of gold, iron oxide, and poly-L-lysine, after which they are easily directed using mild magnetic forces^18^,^19^,^20^,^21^,^22^,^23^,^24^,^25^,^26^,^27^,^28^,^29^. In this assay, these magnetized cells can be rapidly printed using a cylindrical magnet to attract cells to form a spheroid at the bottom of a multiwell plate. The nanoparticles and magnetic forces used to print spheroids are biocompatible, as reported in previous publications^22^,^23^,^27^,^28^,^30^,^31^. In printing the cells, they organize themselves to build a 3D environment that replicates many characteristics of native tissue, particularly cell-cell and cell-ECM interactions. Similar methods have previously been employed to simulate such tissues as fat^23^, lung^27^, aortic valve^28^, blood vessels^21^,^22^, and tumor microenvironments, such as that of breast cancer^29^ and glioblastoma^18^,^19^, all of which show *in vivo*-like protein expression and ECM. From this foundation, assays can be developed that take advantage of the representative environment of spheroids.

The general principle of this methodology was first demonstrated in the development of a 3D analog to the scratch assay^26^, wherein magnetized cells were printed into a 3D ring. Cells interacted with each other and ECM to close the ring as a function of cell migration and proliferation, thereby mimicking wound healing^26^. With M3DB spheroids, building on the principles of the aforementioned ring assay, a novel assay was developed utilizing spheroid size and its change because of early events, like compaction^32^,^33^,^34^, as an endpoint for cytotoxicity. This assay represents an expansion of application into general cytotoxicity from the ring assay. After rapidly generating M3DB spheroids and removing the magnetic field, spheroids immediately contract in size, as cells rearrange and compact to find an equilibrium size from which to grow long term (Fig. 1)^32^,^33^,^34^. When compounds are added immediately after printing, the rate of contraction is varied in a dose-dependent manner, as measured by the change in the projected area over time. In the presence of toxic compounds, spheroids contract at a slower rate. Given the large size of spheroids and their contrast with media due to their brown color imparted by the nanoparticles, their change in size can be imaged at programmed intervals using a mobile device. As a result, spheroids can be imaged simultaneously in a multiwell plate, rather than individually under a microscope, thereby improving imaging throughput and efficiency^26^. Thus, spheroid contraction is a unique phenomenon that occurs immediately after printing which this assay capitalizes on as an endpoint for cytotoxicity for rapid testing and imaging.

The goal of this study was to validate spheroid contraction as a measure of cytotoxicity using 3T3 murine embryonic fibroblasts, following the recommendations from the National Institute of Environmental Health Sciences for the development and validation of cytotoxicity assays^35^. To validate this assay, spheroid contraction was measured using a panel of seven compounds: all-trans retinoic acid (ATRA), 5′-fluorouracil, dexamethasone, doxorubicin, forskolin, sodium dodecyl sulfate (SDS), and penicillin-G. The positive control for this assay was SDS, a detergent used to denature proteins and lyse cells and a commonly used positive control, and the negative control was penicillin-G, a common antibiotic. Toxic responses in spheroid contraction rate were correlated to viability, cytoskeletal organization, and gene expression within the spheroids. This assay was then compared to the standard methylthiazolyl tetrazolium (MTT) assay performed on 2D cultures, which is commonly used for cytotoxic screens^36^. The results of this study help demonstrate that this assay using M3DB spheroids can overcome the technical limitations in speed, throughput, handling, and imaging of other 3D cell culture platforms to offer a simple yet robust assay for the determination of general cytotoxicity in a 3D environment.

## Results

### Spheroid Toxicity Assay

3T3 cells were successfully printed into spheroids whose dose-dependent contraction was imaged over time using a mobile device-based imaging system. Image analysis showed that over the course of 10 h, the 3T3 spheroids contracted except when exposed to penicillin-G, the negative control (Fig. 2, see Supplemental Movie M1 for a movie of spheroid contraction in response to ATRA). The cytotoxic endpoint was the rate of spheroid size change over the first 150 min. There was a significant separation in contraction (*p* < 0.05) between the vehicle (phosphate buffered saline, PBS) and the high concentration of the positive control (625 μM SDS), from which a Z’ of 0.88 was calculated^37^. Overall, there was a significant (*p* < 0.05) dose-dependent effect on spheroid contraction rate for all compounds from which half-maximal inhibitory concentrations (IC_50_) were calculated, except penicillin-G (Table 1). Overall, spheroids exposed to higher concentrations contracted at a slower rate than spheroids exposed to lower concentrations (see Supplemental Fig. S1-2 for the kinetic and dose-response curves of 3T3 spheroids with other compounds).

### Fluorescent Staining

Spheroids contracted for 72 h were fixed and fluorescently stained to assess viability and cytoskeletal organization (Fig. 2 for 3T3s exposed to ATRA, see Supplemental Figs S3-4 for viability and cytoskeletal staining of spheroids exposed to other compounds). In parallel to the compounds’ effects on contraction, at higher compound concentrations the spheroids were less viable, and exhibited a looser cytoskeletal organization, as demonstrated by the increase in dead cells and the distance between cells.

### Spheroid Contraction v. MTT Assay

The results of the spheroid contraction assay were compared to that of the MTT assay performed in 2D using the same cells and compounds, where cell viability was assessed after 72 h (Fig. 2 for 3T3s exposed to ATRA, see Supplemental Fig. S5 for results with the other compounds). IC_50_ values were indeterminable for 5′-fluorouracil and dexamethasone given that no significant effect of either compound on viability in 2D was found (Table 1). In general, spheroids were more resistant to the toxic effects of the compounds than 2D cultures, with significant differences found between their responses in both environments to ATRA, doxorubicin, dexamethasone, SDS, and penicillin-G (*p* < 0.001).

### Gene Expression Profiling

The gene expression of 3T3s in 3D was profiled and compared with that of 3T3s grown in 2D to elucidate the differences between culture environments (Fig. 3). Of the 364 genes whose expression was profiled, 92 genes had significant differences in expression between 3T3s grown in 2D versus 3D. Of those genes with significant differences, 21 genes were related to apoptosis, with 14 of those genes exhibiting reduced expression in 3D. Within the 8 genes which are related to retinol metabolism, 2 had lower expression levels in 3D. Of the 63 genes related to regulation of the actin cytoskeleton, 51 genes were downregulated in 3D cultures as compared to 2D monolayers.

In parallel, 3T3 spheroids with and without 72 h exposure to 41.6 μM ATRA were profiled to analyze the effect of ATRA in 3D (Fig. 3). A concentration of 41.6 μM was chosen because spheroid contraction was affected at and above that concentration. Gene analysis expression showed that 99 genes were expressed differently between 3T3 spheroids that were exposed to ATRA and those that were not, with 27 genes related to apoptosis, 8 related to retinol metabolism, and 64 related to actin regulation. A total of 40 genes were downregulated as a function of exposure to ATRA, with 15 related to apoptosis, 3 related to retinol metabolism, and 22 related to actin regulation.

In comparison to a published dataset in literature on the effect of ATRA on mice^38^, 11 genes were significantly affected by ATRA both in 3D and *in vivo*, of which 8 had similar effects while 3 had opposite effects between the two environments (see Supplemental Fig. S6). Three of those genes were related to apoptosis, 1 gene was related to retinol metabolism, and 7 of those genes were related to actin regulation.

Between the two comparisons examined in this study (2D versus 3D, in the presence and absence of exposure to ATRA in 3D), a total of 52 common genes showed significant differences in expression, 12 related to apoptosis, 5 related to retinol metabolism, 35 related to actin regulation. Of those genes, a total of 36 genes showed an opposite effect when ATRA was added compared to the effect of transitioning from a 2D to 3D environment, with 7 related to apoptosis, 5 related to retinol metabolism, and 24 related to actin regulation.

## Discussion

**Figure 1:**
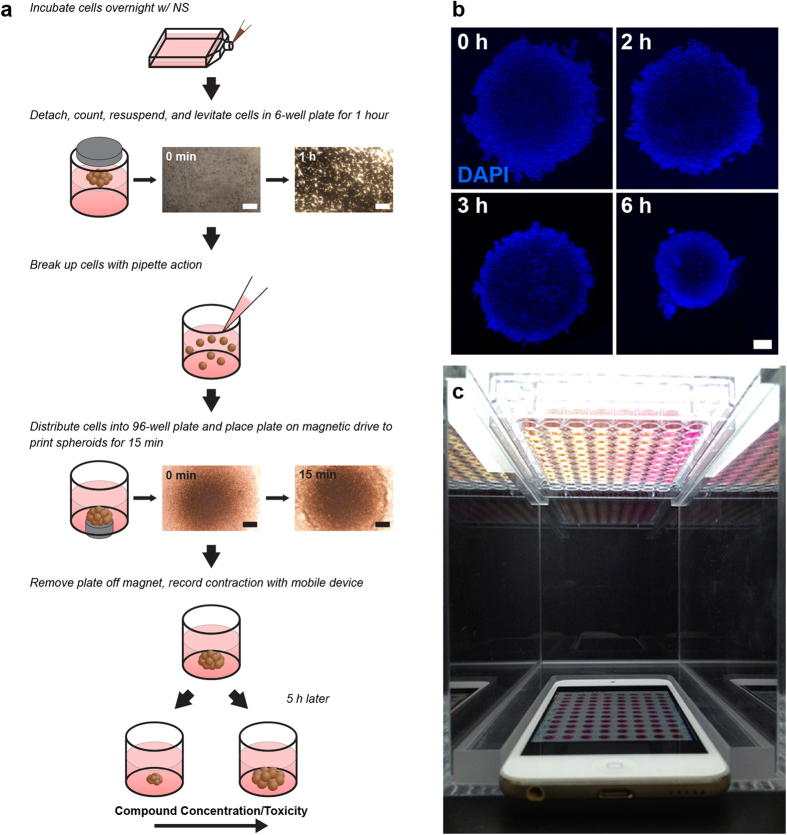
Magnetic 3D bioprinting. (**a**) Cells grown to 70-80% confluence in 2D were incubated overnight with magnetic nanoparticles (NanoShuttle, NS). After resuspending and levitating the cells for a few hours, the cells and ECM were distributed evenly into the wells of a 96-well plate. The cells were then printed for 15 min by putting the plate atop a 96-well magnetic drive. After printing, the magnet was removed and the spheroid contracts. (**b**) Spheroid contraction over 6 h as cells rearrange and compact. (nuclei = blue). (**c**) The mobile device-based imaging system, with the 96-well plate full of spheroids placed above the mobile device, which was set to image the whole plate at programmed intervals as short as 1 s. Scale bar = 250 μm.

**Figure 2:**
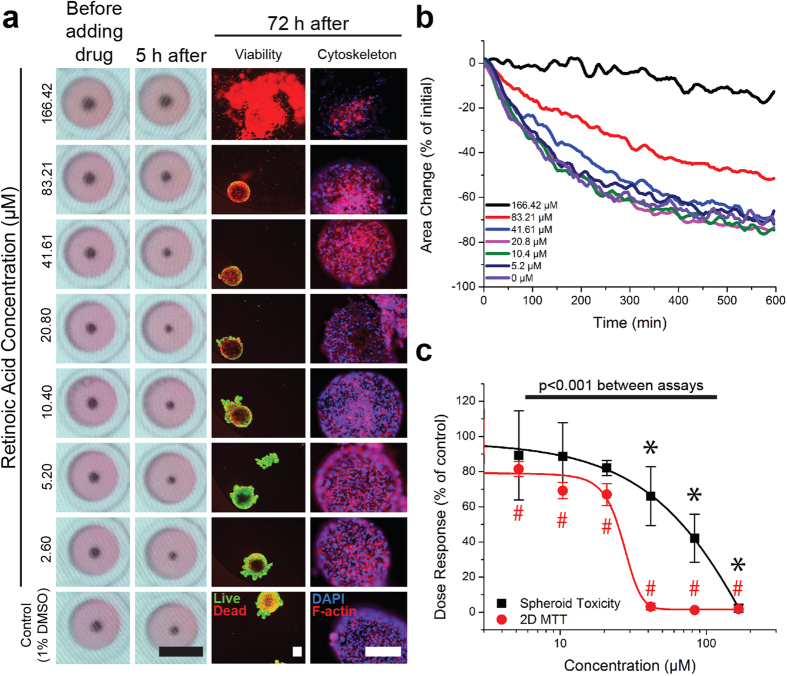
Spheroid contraction of 3T3s in response to ATRA. (**a**) 3T3 spheroids before and 5 h after adding ATRA, as captured by the mobile device (left), live/dead staining (center; live = green, red = dead), and cytoskeletal staining using phalloidin for F-actin (right; F-actin = red, nuclei = blue) of spheroids after 72 h. Black scale bar = 5 mm, white scale bar = 250 μm. (**b**) Kinetics of spheroid contraction. (**c**) The rate of contraction of 3T3 spheroids over 150 min (black) as a function of ATRA concentration and compared to the viability of 3T3s in 2D (red) as measured by the MTT assay. All values are normalized to control. Error bars represent standard deviation. *,#:*p* < 0.05 compared to control. With higher amounts of ATRA, 3T3 spheroids contracted less, were less viable, and showed a more disorganized cytoskeleton.

**Figure 3:**
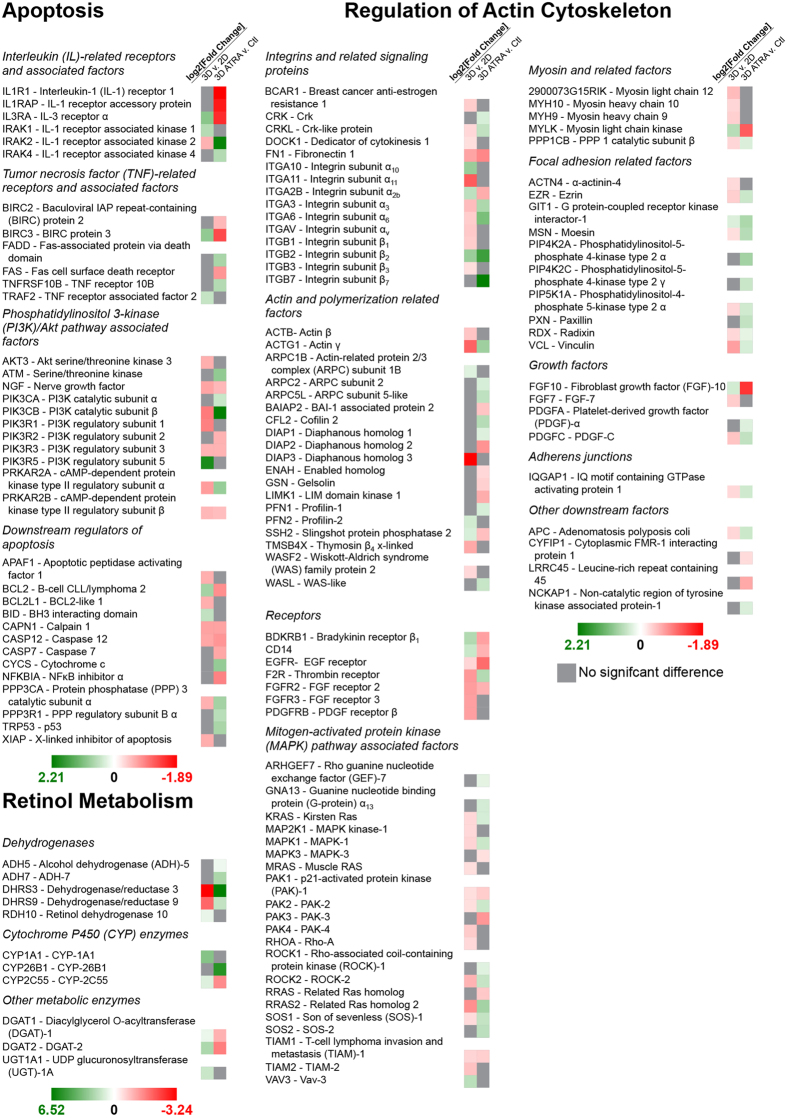
Gene expression profiles of spheroids. Genes related to apoptosis, retinol metabolism, and actin regulation with significant differences (*q* < 0.05) in expression were assessed between 3T3s in 2D or 3D, and 3T3 spheroids in the absence or presence of 41.6 μM ATRA. In transitioning from 2D to 3D, the majority of genes whose expression level significantly changed were reduced, largely in genes related to actin regulation. 41.6 μM ATRA induced upregulation of the gene for p53, a key protein in apoptosis, and downregulation of genes for key apoptotic inhibitors (BCL2, BIRC2, BIRC3), suggesting that exposure to ATRA leads to lesser spheroid contraction via increased apoptosis. Green indicates an increase in gene expression, red indicates a decrease, with darkness indicating the magnitude of change and gray indicating no significant difference.

The goal of this study was to validate contraction of M3DB spheroids as a measure of cytotoxicity. Spheroid contraction is a unique endpoint in which viable cells rearrange and compact immediately after printing as a result of cell-cell interactions to contract the spheroid into a smaller size as a starting point for long-term growth (Fig. 1). When compounds are added at this early stage, the spheroids contract in a dose-dependent manner. There is a relationship between spheroid contraction and cell health, which was confirmed in this study, as M3DB spheroids that contracted less with higher compound concentrations showed lower viability and looser cytoskeletal organization (Fig. 2). 3T3s were also found in this study to be more resistant to compound toxicity in 3D than they were in 2D, with significant differences in gene expression for actin regulation found (Fig. 3). These results validate spheroid contraction as a biologically meaningful endpoint of cytotoxicity in a 3D environment.

The important result of this study is the correlation of spheroid contraction to viability, cytoskeletal organization and apoptosis (Figs 2 & 3). Both SDS (positive control) and penicillin-G (negative control) had their intended effects in either inhibiting contraction or not affecting contraction. A Z’ of 0.88 was found between phosphate buffered saline (PBS, vehicle control) and 625 μM SDS, where values 0.5 ≤ Z’ < 1 indicate a large separation between controls that reflects excellent assay quality^37^. Fluorescent staining revealed that spheroid contraction closely matched viability, similar to previous results in M3DB rings^26^. Gene expression analysis demonstrated differences between spheroids in the presence or absence of ATRA that support findings of toxicity. Specifically, increased expression of p53 (TRP53), a key protein promoting apoptosis^39^, and decreased expression of inhibitors of apoptosis (BCL2, BIRC2, BIRC3)^40^ match previous results on the role of ATRA in inducing apoptosis^41^. Fluorescent staining also revealed an effect of ATRA on cytoskeletal organization, which supports previous results on the effect of ATRA in disorganizing actin^42^. Moreover, ATRA affected a large number of genes that regulate actin. In comparing spheroids to *in vivo* tissue, ATRA had similar effects on 8 genes out of 11 genes significantly affected in both environments (see Supplemental Fig. S6)^38^,^43^. Overall, these results demonstrate that toxic responses, such as from ATRA, can be seen in spheroid contraction, and reflect spheroid viability, organization, and gene expression similar to *in vivo* results^38^.

This study also confirmed that cells in 2D and 3D exhibit significant differences. Cells in 3D had a rounder morphology^4^ (Fig. 1) and a wider array of cell-cell and cell-ECM interactions^3^,^4^,^5^,^6^, which mimic physiological environments. Gene expression profiling showed that a majority of genes related to regulation of the actin cytoskeleton were downregulated in 3D (Fig. 3), likely because cells were not growing on a stiff substrate and retained a more spherical morphology^4^. Such differences were previously reported in magnetically levitated 3D cultures, where cells maintained their phenotype, function, and synthesized ECM^18^,^23^,^28^. Cells in 3D also vary in exposure to molecules between cells on the exterior and cells in the interior, as opposed to cells in 2D, where cells have uniform exposure^44^. This was confirmed in this study when comparing this spheroid assay with the MTT assay, where 3T3s in 3D were found to be more resistant to compounds relative to 2D (Fig. 2), which maybe associated with different exposure to the compound as a function of cellular architecture^44^. The difference in response is stark with penicillin-G, the negative control, which had no effect on spheroid contraction at higher concentrations as expected, but did have an effect on monolayers, albeit at very high concentrations, with an IC_50_ of 5.73 mM listed in the NIEHS registry of cytotoxicity^35^. The disparity in drug resistance is supported by literature, in which only forskolin had a higher IC_50_ in 2D than in this spheroid toxicity assay (Table 1)^16^,^35^,^45^,^46^. The compounds’ IC_50_’s rank similarly between spheroids and cells in 2D in toxicity, but compared to 2D values found in literature, there was a different rank, as forskolin was the most potent compound with spheroids, but dexamethasone was found the be the most potent in literature^45^. Dexamethasone has been shown to reduce expression for the integrin subunit β_2_^47^, and given the increase in expression of ITGB2 in 3D compared to 2D, dexamethasone should have a larger effect on 3D cultures with more β_2_ subunits, and thus the difference in potency rank is reasonable. These results suggest that dimensionality plays a role in compound efficacy, and highlight the importance of assaying compounds in 3D.

What makes spheroid contraction a unique endpoint is that it is simple, biologically relevant, and label-free. Generally, 3D cell cultures are dense in cells and proteins, and data from the center of the spheroid are difficult to capture with reagent-based assays and microscopy^17^. Spheroid contraction escapes these limitations by capturing a culture-wide response through macroscopic imaging without any special reagent or dye. This was demonstrated by the differences between spheroid contraction and viability in 3T3 spheroids measured by the MTT assay, which was unable to resolve differences in any compound except for ATRA, SDS, and penicillin-G (see Supplemental Fig. S7). Moreover, while reagent-based assays typically capture only a single endpoint, spheroid contraction can be captured in real-time, yielding a time-dependent response that could reveal more information about toxic mechanisms or drug interactions. Reagent-based assays or immunostaining can instead be used to supplement spheroid contraction to further explore mechanisms of toxicity, without interference from the magnetic nanoparticles. This attribute was demonstrated in this study with fluorescent staining and gene expression profiling being performed in addition to spheroid contraction. Taken together, spheroid contraction is a robust endpoint for cytotoxicity in 3D, while being amenable to other assays for high-content testing.

Additionally, a mobile device-based imaging system was used in this study for automated image capture. This system is possible given the contrast between the magnetized cells and media (Fig. 2), and the computing power of commercially available mobile devices^26^. A distinct advantage of this system is the ability to image whole plates of spheroids at programmed intervals as small as 1 s, avoiding the need to image individual spheroids under a microscope^26^. With rapid printing, immediate contraction and imaging in less than 3 h, and automated analysis to measure contraction in thousands of images in hours, this assay can be reasonably executed within 24 h. Overall, the mobile device-based imaging system was successfully implemented in this study and demonstrated its efficiency and throughput for spheroid toxicity screening.

Another advantage of this system is the presence of ECM within the spheroid. After levitation and printing, when the compound is added and the magnet is removed, the cells are loosely connected with ECM. This was demonstrated by the presence of laminin and fibronectin in the spheroid, and their growth with longer levitation (see Supplemental Fig. S8) and printing times (see Supplemental Fig. S9-10). The spheroids were dense enough to respond differently than cells in 2D. Some cases may require more ECM in spheroids before exposure to toxic compounds, for which spheroids can be left on the magnet for longer to allow spheroids to build ECM (see Supplemental Fig. S9-10). Thus, levitation and printing can be varied to allow for more mature spheroids.

The spheroid toxicity assay of this study is based on a previously published assay using similar workflows and imaging to assay 3D rings made using magnetic 3D bioprinting^26^. This spheroid assay builds on the ring assay in several ways. First, the main difference between the ring and spheroid assays is the shape, wherein spheroids require far less cells (7.5 × 10^4^ cells/spheroid v. 2 × 10^5^ cells/ring). In requiring far less cells, the spheroid assay improves upon the cost of the ring assay, and can be more easily extended to higher throughput formats (384- and 1536-well plates). These different shapes also model different scenarios. Ring closure is akin to a scratch assay, where cell monolayers are injured with a circular void or linear scratch that cells will close over time^48^, and could similarly be used to assay wound healing responses in 3D. Spheroid contraction expands on the ring assay’s application to model general cytotoxicity, and the ability of cells to migrate, interact with other cells and compact to form the spheroid. Thus, while this spheroid assay carries some of the general attributes as the ring assay, its modeling of general cytotoxicity and high-throughput format improves upon the ring assay and further addresses unmet needs in this field.

In conclusion, this study used M3DB to rapidly print and assay spheroids, which contract as a function of migration, compaction, and cell-cell interactions. Spheroid contraction was tracked using a mobile device-based imaging system, and was validated as a measure of cytotoxicity. The minimal workflow of this assay meets the demand for simple endpoints in high-throughput compound screening. Moreover, immunohistochemistry and genomic analysis demonstrated that these spheroids are amenable to high-content testing. The resulting assay offers a simple, rapid, and robust endpoint for high-throughput cytotoxicity screening in representative 3D environments.

## Materials and Methods

### Cell Culture

3T3 murine embryonic fibroblasts were cultured in Dulbecco’s Modified Eagle Medium (ScienCell, Carlsbad, CA) with 10% fetal bovine serum (Access Biologicals, Vista, CA) and 1% penicillin/streptomycin (Sigma-Aldrich, St. Louis, MO). Cells were cultured in a humidified environment (37°C, 5% CO_2_) with media exchanged every other day.

### Compounds

The following compounds were tested in this study (all purchased from Sigma-Aldrich): ATRA, 5′-fluorouracil, forskolin, doxorubicin, dexamethasone, SDS, and penicillin-G. SDS and penicillin-G served as the positive and negative controls, respectively, for this assay. The compounds were prepared in either (maximum final concentration in parentheses): 1% dimethyl sulfoxide (DMSO, Sigma-Aldrich) for ATRA (166.42 μM) and dexamethasone (509.60 μM); or PBS (pH~7.4, Sigma-Aldrich) for doxorubicin (100 μM), 5′-fluorouracil (256 μM), forskolin (10 μM), SDS (625 μM), and penicillin-G (5.98 mM). Vehicle controls were exposed to the solvent alone and tested for each compound, with its analysis being conducted separately from that of other vehicle controls with the same solvent.

### Spheroid Toxicity Assay

The spheroid toxicity assay was designed to rapidly assess toxicity in M3DB spheroids (Fig. 1A)^26^. At 70–80% confluence, 3T3s grown in 2D were statically incubated overnight with magnetic nanoparticles, allowing for nanoparticle association with the cells (NanoShuttle, Nano3D Biosciences, Houston, TX) at a concentration of 1 μL/1 × 10^4^ cells (50 pg/cell)^18^,^25^. Cells were magnetized by the electrostatic and non-specific attachment of nanoparticles to the cell membrane via poly-L-lysine. Once attached onto the membrane, the nanoparticles remain for 7–8 d before releasing into the ECM^18^. If internalized, the nanoparticles will exit the cell still attached to the membrane. These nanoparticles, along with the magnetic fields, have been shown to have no effect on cell proliferation^18^,^22^,^23^, viability^26^, metabolism^27^,^28^, and inflammatory^27^ and oxidative^28^ stress.

After magnetization, the cells were enzymatically detached with trypsin, resuspended in media, then distributed into ultra-low attachment 6-well plates (Corning, Tewksbury, MA) at a concentration of 3.2 × 10^6^ cells in 2 mL (1.6 × 10^6^ cells/mL). A magnetic drive of 6 neodymium magnets (Nano3D Biosciences) was placed atop the well plate to levitate the cells in 3D (500 G, 30 pN, see Supplemental Fig. S12). The purpose of this levitation step was to induce ECM formation, as was shown previously with primary human pulmonary fibroblasts and tracheal smooth muscle cells that extruded laminin after 1 h of levitation^27^. Moreover, immunohistochemical staining for fibronectin and laminin in 3T3s with varying levitation times demonstrates that the longer 3T3s levitate, the more ECM is produced (see Supplemental Fig. S8-10). After 1 h of levitation, the levitated cultures were broken up and resuspended in media using pipette action to yield a suspension of magnetized cells and ECM, which was distributed into an ultra-low attachment 96-well plate (Corning) at a concentration of 7.5 × 10^4^ cells/well (n = 3 per compound concentration). Immediately afterwards, the plate was placed atop a magnetic drive of 96 neodymium magnets (0.0625” OD, Nano3D Biosciences) to attract the cells (120 G, 10 pN, see Supplemental Fig. S12) and ECM to the bottom of the well to form a spheroid. These spheroids were printed on the magnets for 15 min. At this point, the cells within the spheroids were connected but loose, and their competency could be empirically assessed by their ability to stay together when the magnet was removed. Towards the end of the printing time, compounds were added to the wells achieve their final concentrations. The plate was then removed from the magnet and the spheroids were allowed to contract.

### Mobile Device-Based Imaging and Analysis

M3DB spheroids and their contraction were imaged using a mobile device-based imaging system (Fig. 1B)^26^. After printing, the plate of spheroids were placed atop an acrylic imaging apparatus with a mobile device (iPod touch 5th generation, 16 GB, Apple Computer, Cupertino, CA) below the plate facing upwards, and a light source (LightPad A920, Artograph, Delano, MN) above the plate to illuminate the images. The mobile device was programmed using an application (Experimental Assistant, Nano3D Biosciences) to image the plate every 4 min for a period of 10 h. When the spheroids had finished contracting, the images were transferred to a computer where they were analyzed using a custom image analysis code written in Python that processed thousands of images in hours^26^. The area of the spheroid was measured over time, as well as the rate of contraction for the first 150 min using a linear least-squares fit (OriginPro, OriginLab, Northampton, MA). The rates of contraction served as the endpoint for the assay, and were plotted against compound concentration and fit to a Boltzmann sigmoidal function (OriginPro) from which the IC_50_’s were determined.

### Fluorescent Staining

After spheroid contraction for 72 h, fluorescent staining was performed to assess viability and cytoskeletal organization within the spheroids. Throughout staining, spheroids were anchored to the bottom of the well plate using the 96-well magnetic drive. Spheroids stained for viability (Live/Dead, Biotium, Hayward, CA) were first gently washed with PBS using a multichannel pipet, after which, a solution containing 2 μM calcein AM and 4 μM ethidium homodimer-III was added to each well to incubate for 30 min. The spheroids were then washed again with PBS and imaged under a fluorescent microscope.

Spheroids stained for cytoskeletal organization were fixed with 4% paraformaldehyde (Electron Microscopy Sciences, Hatfield, PA) for at least 5 h. After washing twice with PBS, a solution containing fluorescently-tagged phalloidin (AlexaFluor, Invitrogen, Carlsbad, CA) in PBS was added to each well for 30 min to stain for F-actin. The spheroids were then washed with PBS and counterstained with 4′,6-diamidino-2-phenylindole (DAPI, KPL, Gaithersburg, MD) for 15 min. After one final wash, the spheroids were imaged under a fluorescent microscope.

Spheroids were also stained for nuclei to visualize contraction after printing. Spheroids were printed and fixed with 4% paraformaldehyde 0, 1, 3, and 6 h after printing for at least 5 h. After washing twice in PBS, the spheroids were stained for nuclei with DAPI for 15 min, then imaged under a fluorescent microscope.

### MTT Assay

The MTT assay was performed to compare its endpoint, viability of 3T3s in 2D after 72 h, with spheroid contraction. 3T3s were seeded on 96-well plates (n = 3 per compound concentration) at a concentration of 2.5 × 10^4^ cells/well to adhere overnight. The following day, compounds were added to each well at the desired concentration. After 72 hours of incubation, the media was replaced with 10% MTT (Sigma-Aldrich) in media. The cells were incubated with the reagent for at least 4 hours at 37 °C. Following incubation, the media was aspirated and the remaining formazan was dissolved in acidified isopropanol (0.1 N HCl). The absorbance of the solution was then read at 570 nm in a spectrophotometer (SpectraMax Plus 384, Molecular Devices, Sunnyvale, CA) with background subtraction at 690 nm. Dose-response curves were plotted and fit to a Boltzmann sigmoidal function (OriginPro) similarly to the spheroid toxicity assay.

### Gene Expression Profiling

3T3 spheroids were exposed to 41.6 μM ATRA and contracted for 5 h, then snap frozen in −20 °C overnight to lyse the cells. 3T3s in 2D were also cultured for 72 h and frozen. 3T3s in 2D exposed to 41.6 μM ATRA lysed before 72 h and thus RNA could not be isolated from those samples. Total RNA was isolated from the frozen samples of (RNeasy Mini kit, Qiagen, Venlo, Netherlands) following manufacturer’s instruction without on-column DNase treatment. Magnetic nanoparticles were removed in this process as previously described in literature^28^. mRNA sequencing was conducted for gene expression profiling. The RNA library of each sample was prepared using a directional mRNA-Seq sample preparation kit (Illumina, San Diego, CA). Single-end reads of 50 nt were generated for each sample (HiSeq 200, Illumina). The reads were mapped to genomes (hg19, mm9, and rn4 respectively) using Tophat v1.4.1^49^ and assembled with Cufflinks v2.2.1^50^.

### Statistical Analysis

Statistical analysis for spheroid contraction and the MTT assay was performed with one way analysis of variance (ANOVA) tests (OriginPro). *Post hoc* Tukey’s testing was performed to observe groupwise comparisons for those compounds with significant effects of concentration. The Cuffdiff module of Cufflinks was used for differential expression analysis^51^. Specific categories of genes (apoptosis, retinol metabolism, and actin regulation) were selected to generate the heatmap. Significance was defined as *p*, *q* < 0.05.

## Additional Information

**How to cite this article**: Tseng, H. *et al.* A spheroid toxicity assay using magnetic 3D bioprinting and real-time mobile device-based imaging. *Sci. Rep.* **5**, 13987; doi: 10.1038/srep13987 (2015).

## Supplementary Material

Supplementary Information

Supplementary Movie M1

## Figures and Tables

**Table 1:** IC_50_ values of compounds tested on 3T3s using the spheroid toxicity assay, or in monolayers using the MTT assay.

**Compound**	**3D**	**2D**	
	**Spheroid Assay IC_50_ (μM)**	**MTT IC_50_ (μM)**	**Literature IC_50_(μM)**
*Endpoint*	*Rate of contraction over 150* *min*	*Viability at 72* *h*	
ATRA	82.8	24.7	20 (Seiler *et al.*, 2011)^16^
Dexamethasone	84.6	n.s.	2.77 × 10^−3^ (Nakada *et al.*, 1987)^45^
Doxorubicin	46.7	12.7	0.33 (NIEHS Registry of Cytotoxicity)^35^
5′-fluorouracil	253	n.s.	2.6 (NIEHS Registry of Cytotoxicity)^35^
Forskolin	4.7	0.6	26 (Varrault *et al.*, 1992)^46^
***SDS (positive control)***	281.8	167.2	270 (NIEHS Registry of Cytotoxicity)^35^
***Penicillin-G (negative control)***	n.s.	1769.3	5730 (NIEHS Registry of Cytotoxicity)^35^

These results were then compared to those IC_50_ values, reported in literature^16^,^35^,^45^,^46^. n.s. = not significant.
