# Supplementary material for: A spheroid toxicity assay using magnetic 3D bioprinting and real-time mobile device-based imaging
Source: Sci Rep. 2015 Sep 14;5:13987. doi: 10.1038/srep13987 (PMC4568483; doi:10.1038/srep13987)
Supplement: Supplementary Information [file srep13987-s1.doc]

**A spheroid toxicity assay using magnetic 3D bioprinting and real-time mobile device-based imaging - *Supplemental Data***

Hubert Tseng, Ph.D.^1^, Jacob A. Gage, B.S.^1^, Tsaiwei Shen, Ph.D.^2^, William L. Haisler, M.B.E.^1^, Shane K. Neeley, M.B.E.^1^, Sue Shiao, B.S.^1^, Jianbo Chen, M.S.,^3^ Pujan K. Desai, B.S.,^1^ Angela Liao, B.S.,^1^ Chris Hebel, B.S.^2^, Robert M. Raphael, Ph.D.^1,4^, Jeanne L. Becker, Ph.D.,^1^ Glauco R. Souza, Ph.D.^1,*^

^1^Nano3D Biosciences (n3D), Houston, TX 77030 USA

^2^LC Sciences, Houston, TX 77054 USA

^3^Department of Physics, Rice University, Houston, TX 77005 USA

^4^Department of Bioengineering, Rice University, Houston, TX 77005 USA


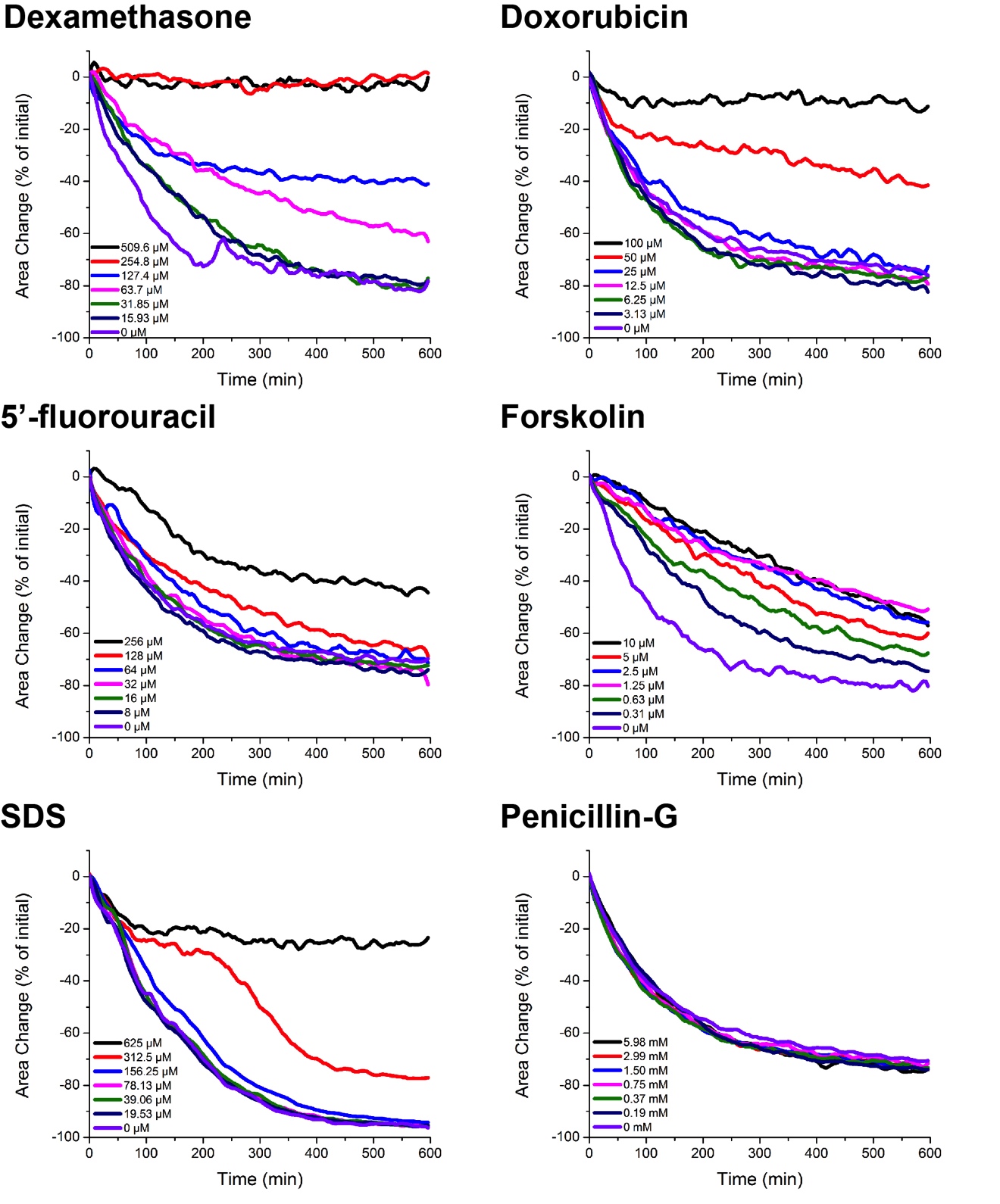


Figure S1: Kinetics of 3T3 spheroid contraction when exposed to either dexamethasone, doxorubicin, 5'-fluorouracil, or forskolin. Note that with increasing drug concentration, the spheroids are unable to contract as quickly as control.


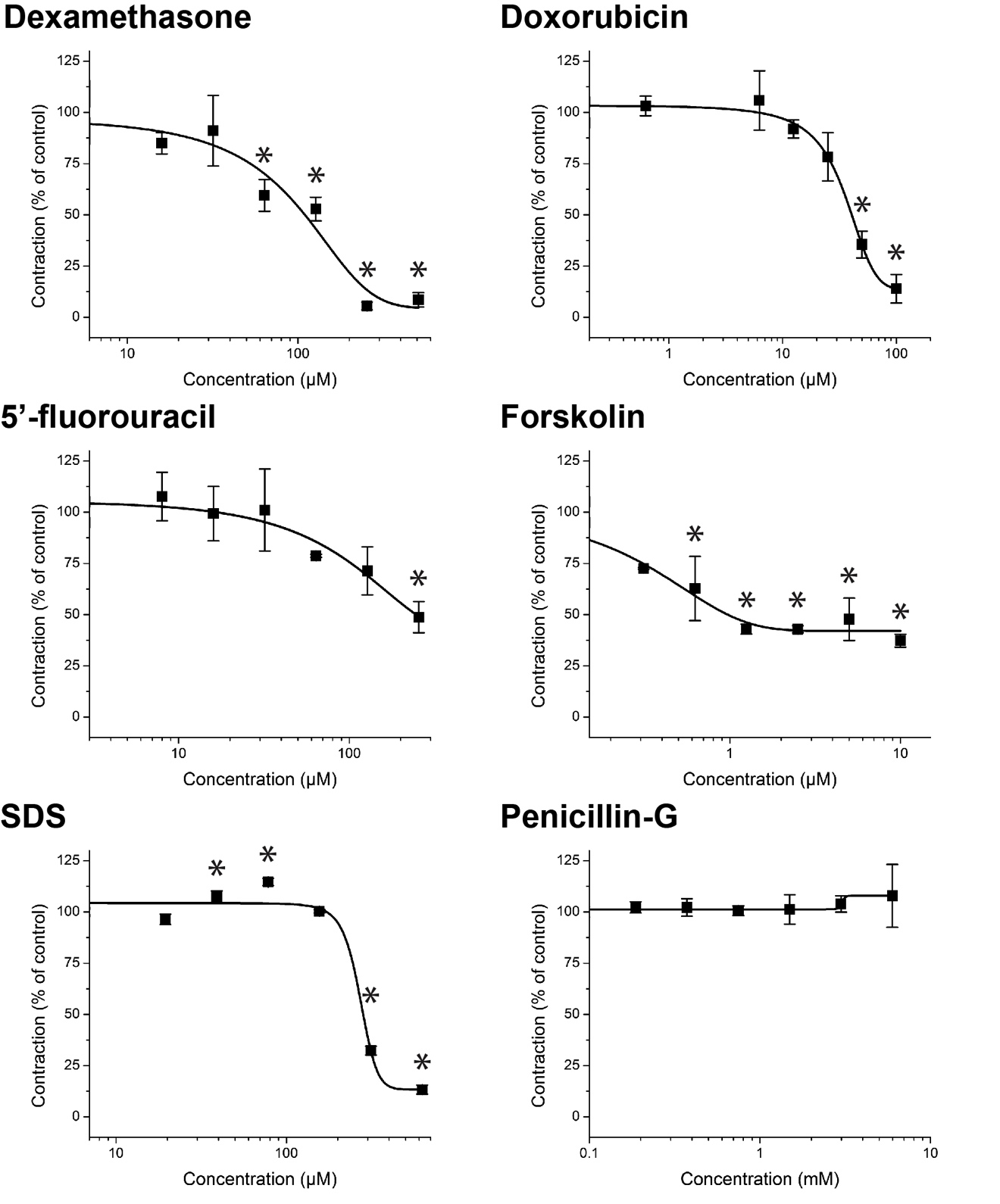


Figure S2: Dose-response curves of 3T3 spheroid contraction when exposed to either dexamethasone, doxorubicin, 5'-fluorouracil, or forskolin. Spheroids exposed to higher drug concentrations are unable to contract as quickly as control. All rates normalized to control. Error bars represent standard deviation. *: p < 0.05 v. control.


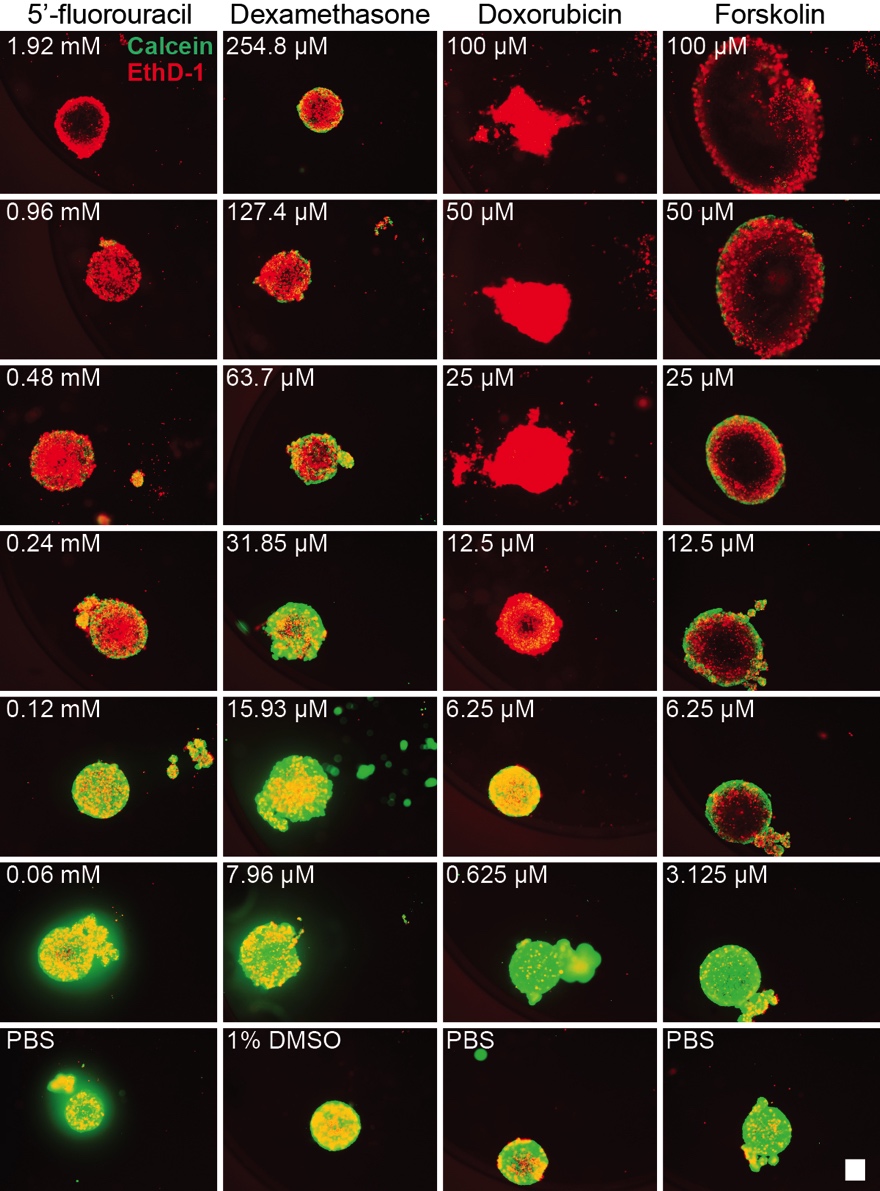


Figure S3: Viability staining of 3T3 spheroids exposed to 5'fluorouracil, dexamethasone, doxorubicin, and forskolin. Green = live, red = dead. Note that with increasing drug concentrations, the cells become less viable within the spheroid. Scale bar = 250 µm.


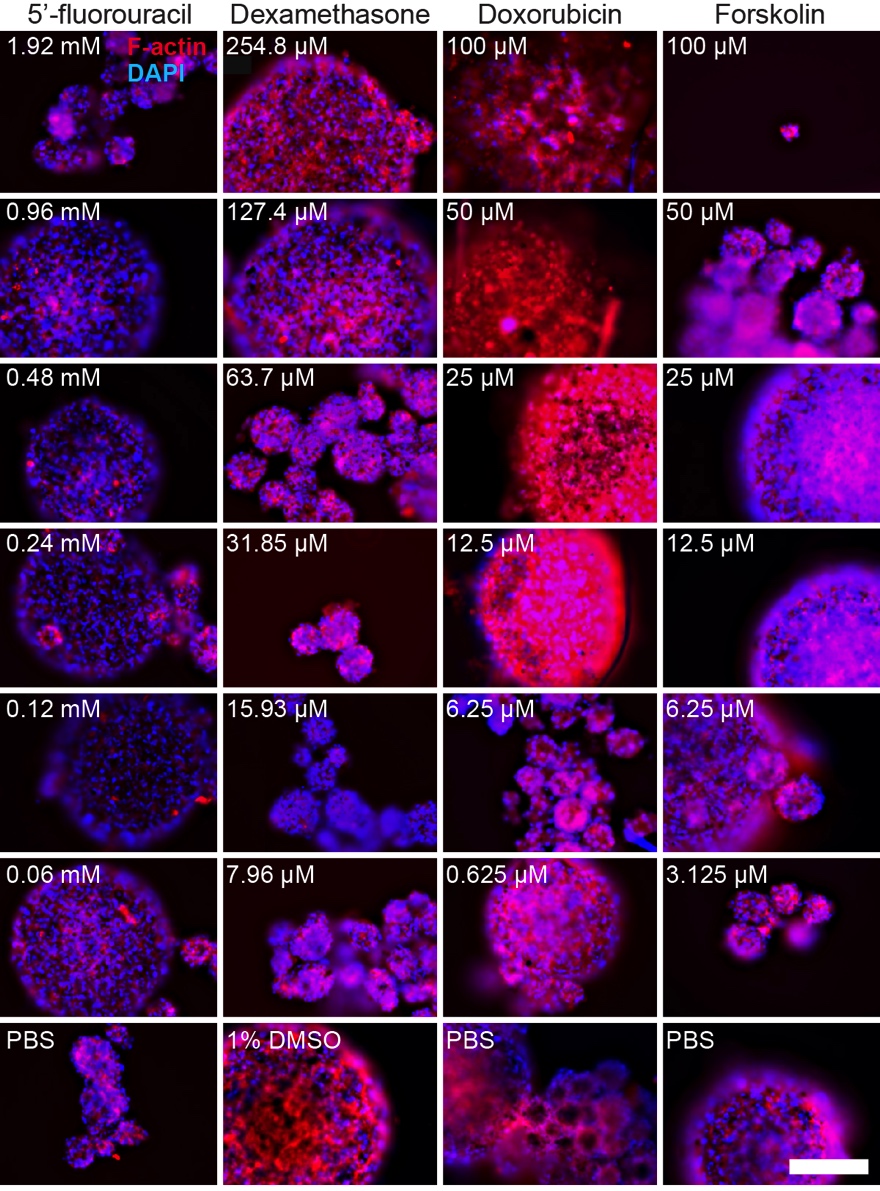


Figure S4: F-actin staining (red) of 3T3 spheroids exposed to 5'fluorouracil, dexamethasone, doxorubicin, and forskolin. Cell nuclei are counterstained with DAPI (blue). Note that with increasing drug concentrations, the spheroids become less organized. Scale bar = 250 µm.


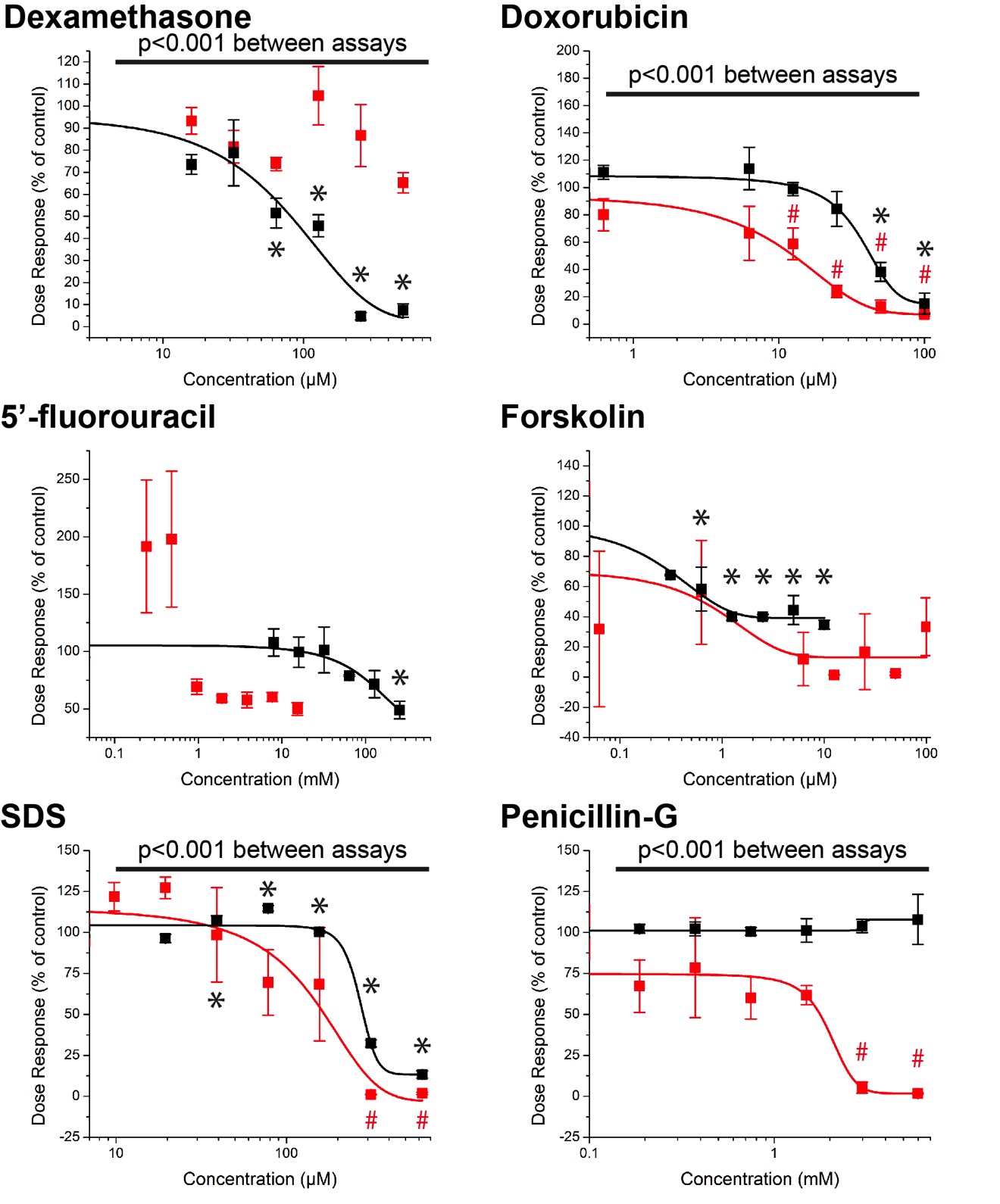


Figure S5: Dose response curves of 3T3s exposed to dexamethasone, doxorubicin, 5'-fluorouracil, and forskolin as measured using magnetically 3D bioprinted spheroids (black) and the MTT assay on 2D cultures (red). In general, spheroids showed a higher resistance to drugs than the same cells in 2D, with a significant effect found with dexamethasone and doxorubicin. *,#: *p* < 0.05 compared to control.


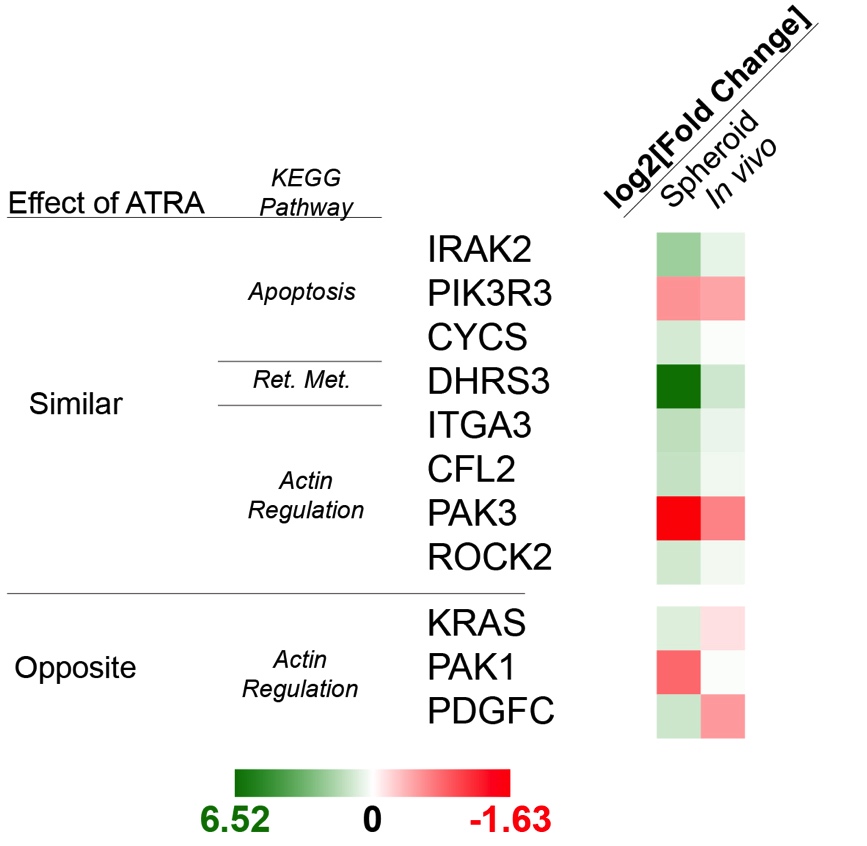


Figure S6: Significant changes in gene expression as a result of ATRA that were commonly found in M3B spheroids and *in vivo*, as reported in literature.^35^ 8 genes were found to have similar effects in both environments, while 3 did not. Green indicates an increase in gene expression, red indicates a decrease, with darkness indicating the magnitude of change.


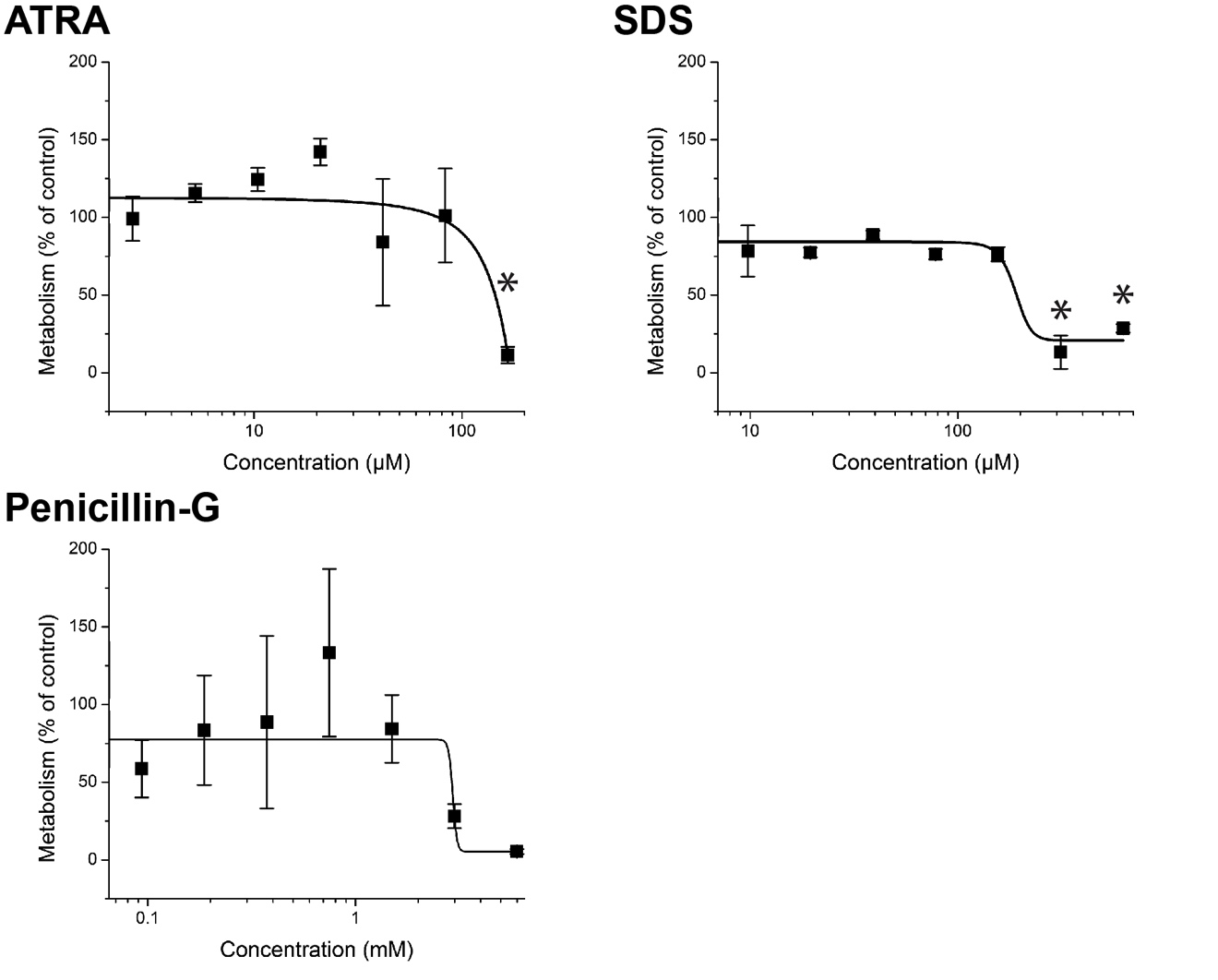


Figure S7: Dose response curves of 3T3 spheroids exposed to ATRA, SDS, and penicillin-G as measured by the MTT assay. Only these drugs exhibited a significant dose-dependent response, suggesting that the MTT assay is insufficient in assaying spheroids, potentially due to the poor diffusion of the MTT reagent through the spheroid.


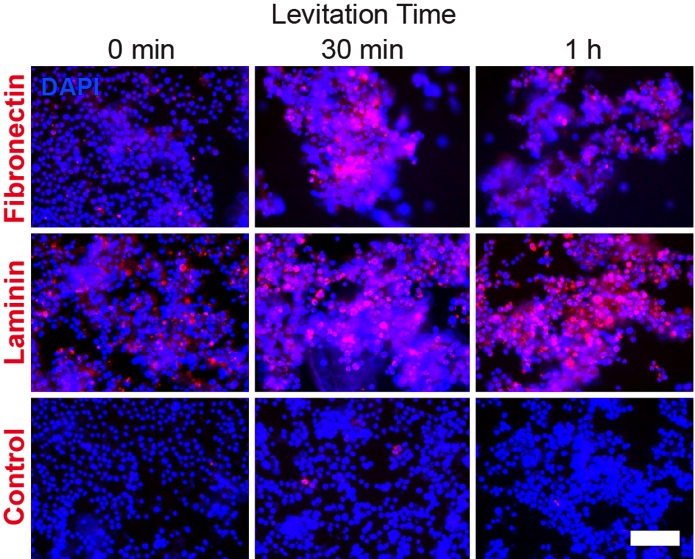


Figure S8: Immunohistochemical stains of levitated 3T3s for fibronectin (red) with varying levitation times. Nuclei are counterstained with DAPI (blue). Within an hour of levitation, 3T3s are extruding ECM in the form of fibronectin and laminin. Scale bar = 100 µm.


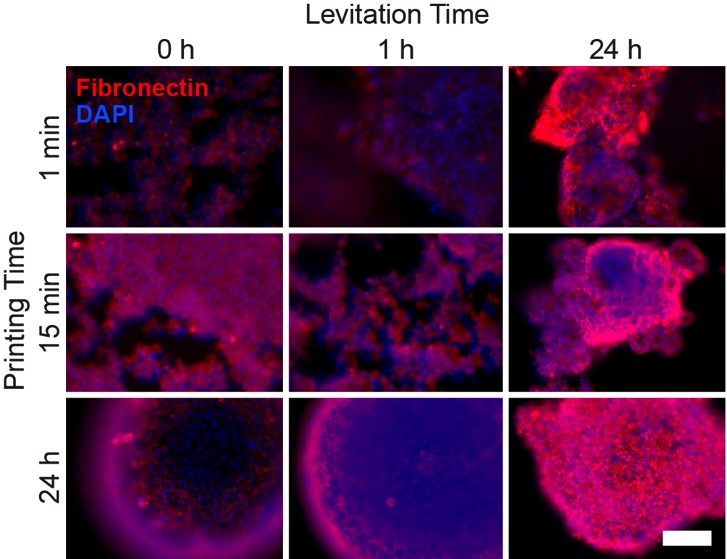


Figure S9: Immunohistochemical stains of 3T3 spheroids for fibronectin (red) that were first levitated and then printed with varying levitation times and printing times. Nuclei are counterstained with DAPI (blue). Fibronectin intensity increased with longer printing times and levitation times. Scale bar = 100 µm.


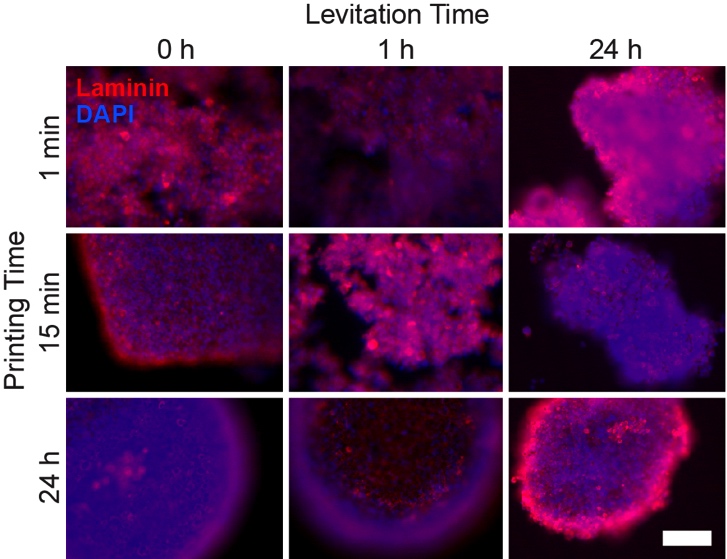


Figure S10: Immunohistochemical stains of 3T3 spheroids for laminin (red) that were first levitated and then printed with varying levitation times and printing times. Nuclei are counterstained with DAPI (blue). Laminin intensity increased with longer printing times and levitation times. Scale bar = 100 µm.


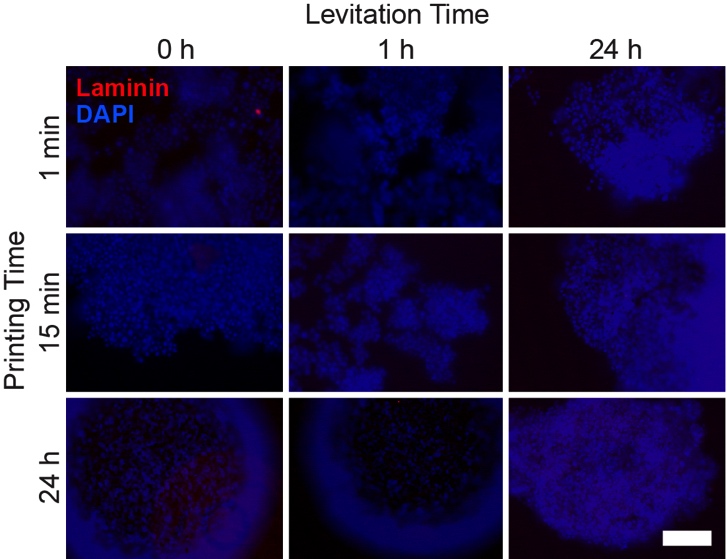


Figure S11: Negative controls for immunohistochemical stains of 3T3 spheroids for laminin and fibronectin that were first levitated and then printed with varying levitation times and printing times. Nuclei are counterstained with DAPI (blue). Scale bar = 100 µm.


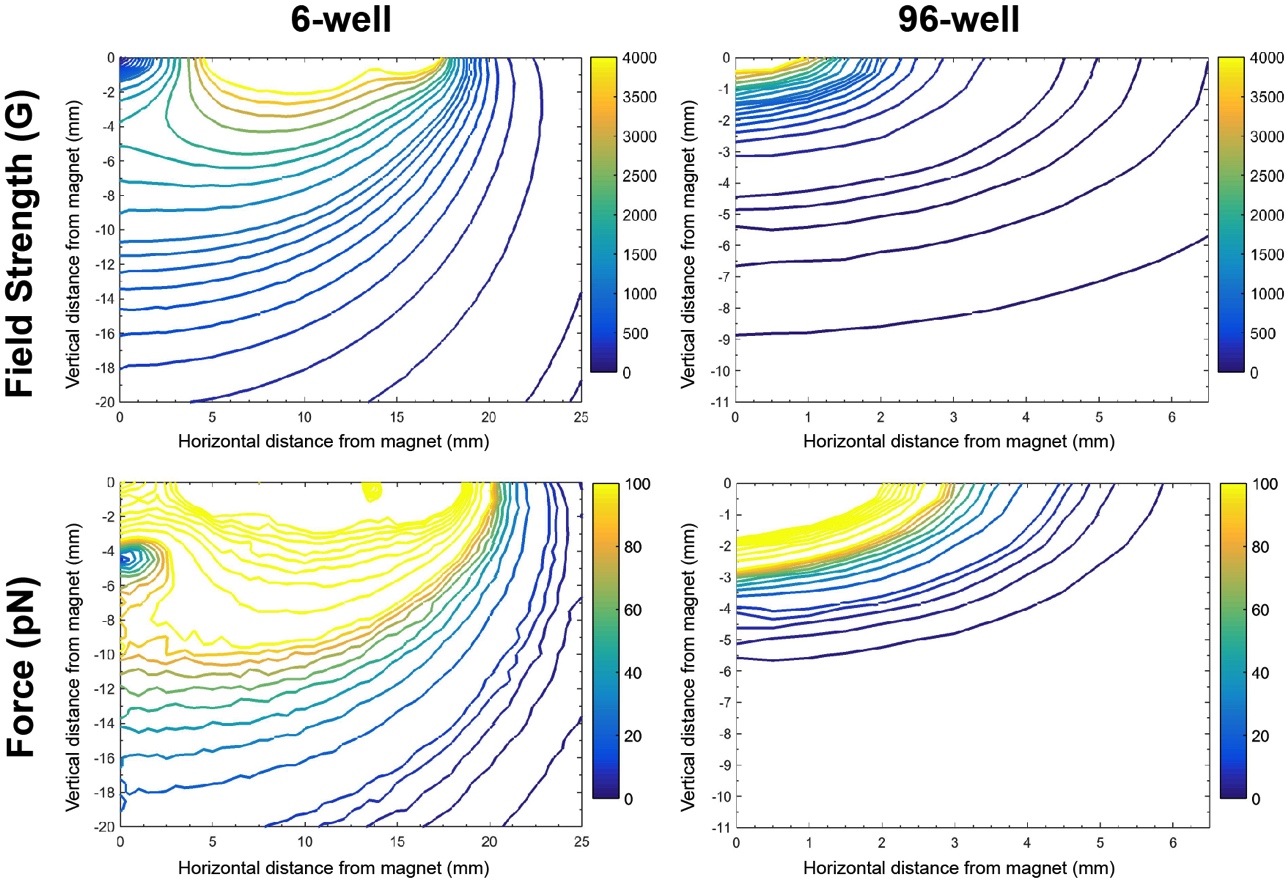


Figure S12: Magnetic field strengths (G) and forces (pN) applied to cells using the 6-well levitation magnet (left) and 96-well bioprinting magnet (right). When levitated under the 6-well magnet, the cells sit 16.35 mm away from the magnet, experiencing a field strength of 500 G (30 pN). When bioprinted with the 96-well magnet, the cells, at 4 mm away from the magnet, experience a field strength of 120 G (10 pN).
